# Nursing and Allied Health Research Priorities in the Care of Patients With Thoracic Malignancies: An International Cross-Sectional Survey

**DOI:** 10.3389/fonc.2020.591799

**Published:** 2020-10-26

**Authors:** Alex Molassiotis, Anne Fraser, Melissa Culligan, Pippa Labuc, Degi L. Csaba, Andreas Charalambous

**Affiliations:** ^1^ School of Nursing, The Hong Kong Polytechnic University, Hong Kong, Hong Kong; ^2^ Blood and Cancer Service, Auckland City Hospital, Auckland, New Zealand; ^3^ School of Medicine, University of Maryland Medical Center, Baltimore, MD, United States; ^4^ Department of Occupational Therapy, Guy’s and St Thomas’​ NHS Foundation Trust, London, United Kingdom; ^5^ Faculty of Sociology and Social Work, Babeş-Bolyai University, Cluj-Napoca, Romania; ^6^ Nursing Department, Cyprus University of Technology, Limassol, Cyprus

**Keywords:** lung cancer, research priorities, nursing, allied health professionals, thoracic malignancies, quality of life, symptoms, interventions

## Abstract

**Background:**

There is currently no evidence of research priorities from nurses and allied health professionals working in the field of thoracic malignancies, which could provide strategic directions for funders, policy makers, and researchers.

**Objective:**

The aim of this study is to identify the priorities for lung cancer and other thoracic malignancies research and practice in nurses and allied health professionals.

**Methods:**

Descriptive cross-sectional web-based international survey conducted through international societies’ membership lists.

**Results:**

Participants included 152 nurses and allied health professionals. Key priority categories were related to developing and evaluation interventions; symptom management interventions; health care system issues; treatment-related research (immunotherapy; targeted therapies); persistent/late effects management (fatigue; pulmonary toxicity); risk reduction, and screening research. The specific topic with the highest endorsement (80.9%) was the development of interventions to improve quality of life. Symptom management interventions, particularly for pain, dyspnea, and fatigue, were also highly endorsed. Health care system topics were related to delivery of care and included nurse-/allied health-led care (67.5%), working with the multidisciplinary team (67.5%), continuity of care (69.2%), and access to care (67.5%). Topics around screening/early detection research were highly endorsed too.

**Conclusion:**

A clear focus (and need) for research in interventions to improve quality of life and symptom management, particularly for pain, dyspnea, and fatigue was also established, alongside healthcare system issues and screening research.

**Implications for practice:**

International societies and funding bodies could consider these topics in their funding decisions and in shaping their strategic directions in the care of patients with thoracic malignancies.

## Introduction

Shifts in cancer care have seen the introduction of more effective treatments such as precision medicine, targeted therapies, and immunotherapy. These novel agents have led to improvements in survival, clinical outcomes, and more focus on prevention, early detection, survivorship, supportive and palliative care. As cancer care changes, so may be the impact of these changes on the patients and their families, creating new or different unmet needs. Nurses and Allied Health professionals need to continue developing new knowledge and addressing clinical unmet needs in order to provide dynamically efficient and patient-centred care. Information on research priorities can provide strategic directions for a particular area of care, highlight a gap in the current knowledge, can be a resource for researchers, policy makers and funding agencies, and potentially can increase the likelihood of research findings influencing clinical practice, care policies, and education. Furthermore, such surveys setting research agendas can elevate the voices of nurses and allied health professionals to shape innovations in care, add value and impact in such innovations by delivering data, creates engaged professionals and allows them to be advocates for their patients, and families’ issues of importance.

Identifying research priorities is often carried out by national or international societies and organisations. For example, the Oncology Nursing Society in USA is conducting research priority surveys almost every four years for the past three decades. Its latest report highlights the priority areas being around patient adherence, screening in minority groups, symptom control, managing late effects, and delivery of survivorship care (1). Other reports focus on specific cancers or specific pathways of care. For example, research in young adults with haematological cancers (n = 80) has identified clinical medicine and psychosocial care as research areas of the highest priority (2). A nurse-patient collaboration project supported by the United Kingdom Oncology Nursing Society (n = 50 nurses and 18 patients) showed a high level of consensus on research related to prevention, screening, early diagnosis, and psychological care across the cancer trajectory (3). Research needs and priorities have been identified in the area of breast cancer (4), kidney research (5), expert panels, or caregivers (6), and prostate cancer survivorship (7) through Delphi consensus. In lung cancer care there is only a small-scale (n = 42) survey of health professionals in Australia, highlighting that reducing the time from presentation of symptoms to diagnosis and treatment was the highest priority while other priorities included timely referral to palliative care or unmet needs in vulnerable populations (8). Another interesting approach to measuring priorities has been the Stakeholder Engagement in quEstion Development and Prioritization (SEED) Method, which is a multi-stakeholder methodology that uses principles of community engagement and causal modelling to develop health research questions that reflect the priorities of patients, clinicians, and other community stakeholder (9). According to the findings of the latter study, the resulting research agenda poses questions on how a broad range of topics including access to care, support systems and coping mechanisms, social determinants of health, and quality of care impacts lung cancer outcomes (9).

The management of lung cancer and other thoracic malignancies has seen significant changes over the past decade with the development of novel therapies, improvements in palliative and supportive care, and earlier diagnosis (5). Also, there is currently no evidence on research priorities from nurses and allied health professionals, which could reflect unmet needs in lung cancer care across the cancer continuum. Hence, the overall aim of the current study is to identify the priorities for lung cancer care research and practice in nurses and allied health professionals. The results from this study can be used to inform the development of lung cancer care-specific research priorities in the wider lung cancer nursing and allied health community and contexts.

## Methods

### Design

This study is a cross-sectional international web-based survey. Survey participants were recruited from the email membership lists of international societies, such as nursing and allied health membership of the International Association for the Study of Lung Cancer (IASLC), International Thoracic Oncology Nursing Forum (ITONF), European Oncology Nursing Society (EONS), Multinational Association of Supportive Care in Cancer (MASCC), and the UK National Lung Cancer Forum(UKNLCF). Individuals participating could have forwarded the survey link to other individuals in their network or even to their national society, as requested through the survey’s information letter. We have also used social media, with the survey being disseminated through Twitter, Facebook, and Linkedin. The survey information letter asked individuals to complete the survey only if they worked exclusively or mostly with lung cancer patients. For nursing, most of the societies were lung cancer specific and hence it was expected that all nursing participants would be working most of their time with lung cancer patients. For allied health professionals, while we left this to be self-defined, we restricted the types of professionals that could participate to a few only by disseminating the survey in societies for occupational and physical therapy, social work, and psycho-oncology only as those work more closely with cancer patients albeit acknowledging this would be a small part of their workload with the exception of psycho-oncology. The sample represents diverse backgrounds in academic and practice settings. The term “lung cancer” in this study reflects patients with any thoracic malignancy. The term “care” includes care provided across the disease trajectory.

### Data Collection

The survey questionnaire on research priorities developed by the Oncology Nursing Society (ONS) (1) was the basis for the questionnaire of this study. Permission was obtained from ONS and the questionnaire was adapted to reflect specific areas of lung cancer care not reflected in the original ONS questionnaire. Questionnaire adaptation was done through discussions with lung cancer care experts and literature on the topic and resulted only in the addition of items not covered in the original ONS survey under the same domains. Six experts (4 nurses, 1 occupational therapist, and 1 psycho-oncologist) also commented on the content, clarity of the questions posed, or wording through two rounds of comments. The web survey was developed through an in-house e-survey platform. The final questionnaire included a section on the participants’ characteristics (sex, age group, society membership, country of residence, professional discipline, years of experience, highest degree, and primary work setting). Questions on research priorities were then broken down into categories/sections, including developing and evaluating interventions (50 statements which also include items on developing interventions for nearly 30 symptoms and 20 complementary therapies), screening research (3 statements), reducing social inequalities in lung cancer care (3 statements), symptom management interventions (with specific focus on 28 symptoms and 3 more general symptom statements), treatment- and diagnosis-related research (14 statements), persistent and late effects (list of 19 late effects), risk reduction in cancer patients and survivors (10 statements), survivorship issues (5 statements), healthcare systems (26 statements), and caregivers issues (12 statements). All statements were rated on a 4-point scale, with “1” representing highest priority and “4” representing not at all of a priority. Participants were then additionally asked to select from a list of 28 symptoms the three most difficult symptoms to manage and the three most distressing symptoms for lung cancer patients. Ethical approval for the conduct of the study was obtained from the Human Research Ethics Review Committee of the Hong Kong Polytechnic University. Email lists were used through society administrators after permission was obtained from the respective chair/president/board. Society members received an email invitation with a letter explaining the purpose of the study, the anonymous nature of the survey, the societies involved, and ethical approval, asking their voluntary participation and stating that completion of the questionnaire would imply consent. A reminder email was sent to the same email lists after 3–4 weeks. The survey was open for four months until late 2019. There was no clear information from most of the societies on the specific number of nurses and allied health professionals, as membership included many different disciplines, and hence no response rate could be calculated. Although there was no predetermined sample size calculation as the population size was not known, as a rule of thumb we expected to have at least 100 responses in order to have any meaningful results.

### Data Analysis

Data analysis was primarily based on descriptive statistics. Frequencies and percentages were calculated for each item of each domain of the survey tool. A rank order of these frequencies was tabulated. The percentage scores refer to proportions of participants who rated the item at a specific priority score (i.e. score 1 for “high priority” to score 4 “low/no priority”). Comparisons were made with regards to education (degree holders or lower vs. postgraduate education) and work setting (inpatient/outpatient/ambulatory setting vs. home care/palliative care vs. educational setting) without the use of any formal statistics.

## Results

### Sample Characteristics

The sample included 152 participants, most of whom (n = 136) were from the nursing discipline. They had an average of 13.4 (SD = 9.8) years of experience working with patients with thoracic malignancies. Most were coming from the USA, UK or other European countries and were members of IASLC, EONS, or ITONF providing an international reach to the required sample. More details are presented in **Table 1**.

**Table 1:** Sample characteristics (n = 152).

		N	%
Gender	Male	24	15.8
	Female	128	84.2
Age group	20–29	9	5.92
	30–39	38	25
	40–49	41	26.97
	50–59	48	31.58
	>60	16	10.53
Country of residence	USA	30	19.7
	UK	21	13.9
	Ireland	14	9.3
	Iceland	9	5.9
	Greece	8	5.3
	Australia	8	5.3
	Cyprus	7	4.6
	Turkey	7	4.6
	Sweden	7	4.6
	Belgium	5	3.3
	Canada	5	3.3
	Europe (other)	19	12.3
	Asia	8	5.3
	Africa	4	2.6
Society membership*	IASLC	47	30.9
	EONS	42	27.6
	ITONF	27	17.8
	NLCFN	9	5.9
	Other society or multiple society membership	49	32.2
Professional discipline	Nursing	136	89.5
	Physiotherapy/Occupational therapy	5	3.3
	Social Work/Psychology	3	1.95
	Others (Speech therapy, Doctor, Pharmacy, Program director, Advocate, Oncocoach)	8	5.25
Highest degree	Associate degree/Diploma	11	7.2
	Bachelor degree	29	19.1
	Master’s degree	72	47.4
	DNP/Professional doctorate	6	3.9
	Doctoral degree (PhD)	34	22.4
Primary place of work	Inpatient care	35	23
	Ambulatory/outpatient care	55	36
	Hospice/palliative care	16	10
	University/College	34	22.1
	Others (research center, home care, day-care, medical center, advocacy, cancer society, government cancer control	12	7.90

*Participants could choose more than one option, hence percentage in higher than 100%

IASLC, International Association for the Study of Lung Cancer; EONS, European Oncology Nursing Society; ITONF, International Thoracic Oncology Nursing Forum; NLCFN, National Lung Cancer Forum for Nurses (UK).

### Research Priorities

Out of the top twenty priorities, the categories of developing and evaluating interventions, symptom management interventions and health care system topics had four specific items selected each. Persistent/late effects, treatment-related research, risk reduction in cancer patients and survivors and screening research had two items selected as priorities each. As the two topics selected in persistent/late effects included symptoms, this combined with the category of symptom management interventions makes the symptoms research as the top priority area. Also, development of interventions in different categories included primarily interventions for symptom control, containing also self-management symptom interventions (69.7%). Looking at specific items selected as top priorities, the highest priority was on interventions to improve quality of life (80.9%). The next two priorities with 78.8 and 73% each were related to interventions for the management of dyspnea and pain, respectively. Other key symptoms that were in the top twenty priority list included fatigue management, and managing pulmonary toxicity and depression (with anxiety management being the 21^st^ topic selected with 61.3%). Palliative care interventions were high in the priority list (72.4%) as was research related to immunotherapy and targeted therapies (around 70%). Health care system topics of high priority included continuity of care, access to care, nurse-led care, and working with the multidisciplinary team. Risk reduction through smoking cessation approaches and screening/early detection, particularly in undeserved and/or uninsured people, accounted for the remaining top priorities. A detailed description of the top twenty priorities is presented in **Table 2**.

**Table 2:** Top 20 Research priorities in lung cancer care.

Rank	Theme	Specific focus	High priority = 1	2	3	Not at all = 4	Mean*	SD
1	Develop and evaluate interventions	Interventions to improve quality of life	123 (80.9%)	25 (16.4%)	3 (2%)	1 (0.7%)	1.22	0.5
2	Symptom management interventions	Dyspnea/Shortness of breath	108 (78.8%)	23 (16.8%)	5 (3.6%)	1 (0.7%)	1.26	0.56
3	Symptom management interventions	Pain (e.g., Chest pain, bone pain)	100 (73%)	32 (23.4%)	4 (2.9%)	1 (0.7%)	1.31	0.56
4	Develop and evaluate interventions	Assistance with management of symptoms	101 (72.4%)	37 (24.3%)	2 (1.3%)	1 (0.7%)	1.29	0.52
4	Develop and evaluate interventions	Palliative care interventions (home/community-based and hospital-based)	110 (72.4%)	37 (24.3%)	2 (1.3%)	1 (0.7%)	1.42	0.67
6	Treatment- and diagnosis-related research	Immunotherapy	92 (71.9%)	30 (23.4%)	6 (4.7%)	0	1.33	0.56
7	Develop and evaluate interventions	Self-management interventions to improve symptom control	86 (69.7%)	36 (23.7%)	7(4.6)	3 (2)	1.39	0.67
8	Treatment- and diagnosis-related research	Targeted therapies	89 (69.5%)	33 (25.8%)	6 (4.7%)	0	1.35	0.57
9	Health care systems	Continuity of care	81 (69.2%)	28 (23.9%)	12 (10.3%)	1 (0.9%)	1.38	0.61
10	Risk reduction in cancer patients and survivors	Smoking cessation	83 (68.6%)	28 (23.1%)	7 (5.8%)	3 (2.25%)	1.42	0.72
11	Health care systems	Access to care	79 (67.5%)	28 (23.9%)	10 (8.5%)	0	1.41	0.64
11	Health care systems	Work with the multi-disciplinary team	79 (67.5%)	28 (23.9%)	7 (6%)	3 (2.6%)	1.44	0.72
11	Health care systems	Nurse-led/AHP-led care	79 (67.5%)	28 (23.9%)	5 (4.3%)	5 (4.3%)	1.45	0.77
14	Persistent and late effects	Fatigue	82 (67.2%)	31 (25.4%)	8 (6.6%)	1 (0.8%)	1.41	0.65
15	Symptom management interventions	Fatigue	91 (66.4%)	41 (29.9%)	5 (3.6%)	0	1.37	0.55
15	Persistent and late effects	Pulmonary toxicity	81 (66.4%)	38 (31.1%)	2 (1.6%)	1 (0.8%)	1.37	0.56
17	Risk reduction in cancer patients and survivors	Screening/early detection	78 (64.5%)	29 (24%)	12 (9.9%)	2 (1.7%)	1.49	0.74
18	Symptom management interventions	Depression	88 (64.2%)	40 (29.2%)	8 (5.8%)	1 (0.7%)	1.43	0.64
19	Screening research	Screening and early detection for lung cancer in underserved and/or underinsured individuals	92 (62.6%)	34 (23.1%)	16 (10.9%)	5 (3.4%)	1.55	0.82
20	Screening research	Screening for lung cancer in at-risk individuals	95 (62.5%)	37 (24.3%)	12 (7.9%)	3 (2%)	1.48	0.73

*Lower mean scores represent higher priority (1 = highest priority, 4 = lowest priority).

The lowest priority (all <20%) was related to all 15 statements about research in different types of complementary and alternative medicine. Other low priority areas, selected by less than 30% of participants, included social support and counselling interventions (30%), interventions that use technology to address symptoms (29.6%), spiritual care (29.6%), bereavement research (28.9%), bio-informatics (25%), and non-medical prescribing (24.8%). In relation to the list of 28 symptoms, the item with the lowest endorsement was unexplained weight loss (38.2%), while cough research was endorsed by 54% of participants.

**Table 3** presents the top ten most difficult to manage symptoms and the most distressing symptoms for patients. Pain, dyspnea, and fatigue were the top three symptoms identified both in terms of difficulty in managing and being distressing for patients. Interestingly, cough, being a common symptom in lung cancer, was 9^th^ in the list of difficult symptoms to manage in the current study, but was recognized as the 4^th^ most distressing symptom for patients.

**Table 3:** Top ten most difficult symptoms to manage and most distressing symptoms for lung cancer patients.

	Difficult to manage symptoms		Distress from symptoms	
	%	Rank order	%	Rank order
Pain (e.g., Chest pain, bone pain)	53.7	1	49.8	1
Dyspnea/Shortness of breath	43.5	2	47.8	2
Fatigue	43.1	3	26.3	3
Functional impairment	16.7	4	17.6	5
Depression	14.9	5	10.8	8
Anxiety	13	6	16.6	6
Cachexia	13	6	5.9	10
Peripheral neuropathy	13	6		
Cough	10.2	9	20.6	4
Cognitive dysfunction	9.3	10		
Sleep/wake disturbances			14.7	7
Immunosuppression-related symptoms			6.6	9

Endorsement of topics was also assessed in terms of highest degree held (Bachelor degree holders and below vs those having postgraduate education) and the work place (inpatient/outpatient/ambulatory setting versus homecare/hospice/palliative care versus university/college setting). Regarding education level, the key priorities were consistent between the two groups, with symptom management and quality of life being the common priorities. The group with baccalaureate education and below was further concerned on access to care, whereas those with postgraduate education highlighted research in immunotherapy as a key priority for them. In terms of priority endorsement based on work setting, symptom management interventions and interventions to improve quality of life were also common across all three groups. However, the hospital-based group prioritized other clinical topics (i.e. management of pain and dyspnea and immunotherapy research), the community/palliative care group had additional emphasis on psychosocial adjustment, while the education-based group had additional emphasis on self-management interventions and health care system aspects such as continuity of care and access to care (**Table 4**).

**Table 4:** Differences and similarities in research priorities based on education and work setting.

Participants with BSc/Diploma			N (%)	Participants with MSc, DNP, PhD			N (%)
Interventions to manage Pain (e.g., Chest pain, bone pain) Interventions to manage Dyspnea/Shortness of breath Pulmonary effects Access to care Interventions to improve quality of life			27 (87.1%) 25 (80.6%) 19 (79.2%) 19 (82.6%) 32 (80%)	Interventions to improve quality of life Intervention to manage Dyspnea/Shortness of breath Intervention to manage Pain (e.g., Chest pain, bone pain) Assistance with management of symptoms interventions Immunotherapy			94 (83.9%) 79 (79.8%) 74 (74.7%) 81 (73%) 64 (69.6%)
**Participants from inpatient/outpatient/ambulatory care**	**N (%)**	**Participants from homecare/hospice/palliative care**			**N (%)**	**Participants from universities/colleges**	**N (%)**
Interventions to improve quality of life Intervention to manage Dyspnea/Shortness of breath Intervention to manage pain Immunotherapy Assistance with management of symptoms interventions	74 (82.2%) 63 (79.7%) 59 (74.7%) 52 (73.2%) 63 (70.8%)	Intervention to manage pain Persistent and late effects (Pulmonary) Psychological adjustment and coping Assistance with management of symptom interventions Interventions to improve quality of life			14 (93.3%) 11 (91.7%) 10 (90.9%) 15 (88.2%) 14 (82.4%)	Continuity of care Assistance with management of symptoms interventions Access to care Self-management interventions to improve symptom control Interventions to improve quality of life	23 (76.7%) 23 (69.7%) 22 (73.3%) 26 (76.5%) 25 (73.5%)

## Discussion

This is the first survey of nursing and allied health professionals focusing on their research priorities in the field of thoracic malignancies. Key priorities were about developing interventions to improve quality of life, symptom management, and palliative care. Endorsements of high priority also included health care system-related research reflecting issues around the delivery of care, treatment-related research (immunotherapy and targeted therapy), persistent/late effects management of pulmonary toxicity and fatigue, smoking cessation as a way to reduce risk in patients and screening/early detection research. Pain, dyspnea, and fatigue were the highest ranked symptoms both in terms of difficulty in managing them and the distress impacting upon patients.

The focus on development and evaluation of interventions to improve quality of life and symptom management reflects the significant unmet needs of patients with lung cancer, who are often diagnosed at a late stage experiencing at the same time a complex array of supportive care needs, while our knowledge on how to manage these needs is fairly fragmented (10). This is also an area of care that has produced new challenges as a result of the introduction of newer treatments with complex and difficult symptoms to manage (11). Pain was endorsed as the most difficult symptom to manage, perhaps reflecting more complex pain syndromes in largely palliative care patients where the evidence-base is limited and the research investment minimal. Dyspnea has received more research attention over the years, but still our knowledge is not adequate to provide complete relief to patients. However significant efforts in finding new interventions continue and new approaches are developed (12, 13). Managing (refractory) fatigue is a topic featuring at the top of complex, distressing and difficult to manage symptoms for decades now across cancer groups, and was also identified as the most difficult symptom to manage and the most distressing for patients in the ONS 2013 survey (1). A number of interventions, primarily non-pharmacological ones, have shown promising results for several symptoms (14, 15), although the uptake of such approaches in clinical practice is often less than optimal. Pulmonary toxicity has received high endorsement as a key research area, not only reflecting perhaps the frustration of clinicians in managing this difficult symptom but also as an example where a multidisciplinary effort is needed in order to provide optimal care, connected with the health care systems related topic in the survey.

Cough is a symptom that 57–67% of patients with lung cancer experience (16) and is severe enough to require treatment in as many as 62% of them (17). The complexity of its treatment is also highlighted in the most recent clinical guidelines developed by the American College of Chest Physicians (18). However, it was not endorsed by our sample as a key research priority on symptoms, although it was recognized as the fourth most distressing symptom for patients. A possible interpretation of this finding lies in the fact that lung cancer-related cough is an important unmet clinical need for which morbidity and distress are often underestimated by health professionals (16). This discrepancy needs to be elucidated a little more clearly in the future.

Psychosocial care topics received low endorsement generally, including coping, psychosocial adjustment, bereavement care, and spiritual care, with the exception of managing depression. Only those participants working in the community and palliative care settings endorsed these higher than the rest of the participants. Psychosocial care is key to improving quality of life, and often a high priority area in many past surveys (1–3). It would be useful in the future, perhaps with qualitative research, to explore this discrepancy further in the lung cancer field.

Delivery of care and health care system-related issues have been the focus of nursing and allied health for a couple of decades with the identification and evaluation of service provision, service models and early palliative care, reviewed elsewhere (11). The changing face of cancer care is an area where the specialized roles across nurses and allied healthcare professionals become pivotal (19). The rise of new treatments and consequently of new and often complex adverse events (e.g. irAEs) requires specialized training and skills in order to timely diagnose, treat, and monitor over time (20). Furthermore, as the needs of patients change there are also opportunities to deliver care in a more patient-centred and optimal way. Novel targeted therapies have led to increased survival in some of the lung cancer population, opening the discussions around survivorship care in this population. To achieve appropriate delivery of often complex care in lung cancer, three issues from the health care system topics that ranked the highest are important to consider, including a) nurse/allied health-led care, b) continuity of care rather than fragmented care as we currently see in many places (11, 21) and c) the role of the multidisciplinary team. Access to care continues to be of concern, similarly to other nursing surveys (1). Some topics in this category received low endorsement, such as non-medical prescribing, which may not be necessarily related to lack of research interest but rather with the perception that the topic has been covered already and there is enough data on evidence or delivery issues and further work may not be a priority at this stage. Furthermore, treatment-related research was identified in this sample of high priority, including immunotherapy and targeted therapies. These therapies are changing the treatment field in lung cancer and hence provide hope for many and the participants recognized that more research in optimising these novel treatments is necessary.

An interesting finding was the lowest priority attributed to all the 15 statements about research in different types of complementary and alternative medicine. This finding comes in contrast to studies that demonstrate an uprising in the numbers of patients with cancer (including lung cancer patients) who choose to utilize CAM and CAM use is reported in 42% of lung cancer patients (22). The frequent use of CAM within the lung cancer context is notable and there is a need for obtaining information on their use, particularly in controlled clinical trials, to prospectively document it.

There is a strong case for more research in screening/early detection for lung cancer (23). However, specifically for nursing, in a recent systematic review it was demonstrated that only a small fraction of studies was attributed to this field of care across cancer types (20). As most patients with lung cancer are diagnosed at a late stage, where cure is not an option, the participants emphasized that screening and early detection alongside with smoking cessation to reduce risk is highly desirable. Screening/early detection in at risk populations such as minorities and underserved and uninsured populations in the wider cancer filed were also the third and fifth highest priorities in the ONS 2013 survey too (1). Promising work in the field of early detection highlights that such approaches may be linked with enhanced clinical outcomes and potentially be cost-effective (24, 25).

Strengths of this survey include efforts to represent international perspectives; adaptation of an existing established survey as a base; intended breadth and inclusiveness of survey items by including multiple facets of care; and unique focus on lung cancer specifically. Limitations of this survey are similar to any web-based surveys, including difficulty in establishing a representative sample and difficulties with reach. While a response rate for this survey was not established due to the lack of separate categories available in email lists of large international societies, response rates in similar surveys are typically very small. Indeed the ONS 2013 survey (1) had a response rate of 11%, similar to previous ONS surveys. While every effort was made to encourage allied health professionals to participate and several related societies were approached, either there was no response from the societies or minimal response from their members (who often do not work exclusively in cancer care), leading to a very small number of allied health professionals participating. Hence, there was lack of specificity in “nursing” and “allied health professional” inclusion criteria and the data from this survey reflect more the views and priorities of nurses. In the future, more targeted sampling for allied health professionals will be necessary. Finally, there was lack of differentiation between individual survey items; this may have led to some confusion or difficulty in the interpretation of the items by the respondents, although the domain title for each of these items, which was visible to respondents, provided some context for them to consider before replying.

## Conclusions

There is strong support from the data presented that future research should focus on the development and evaluation of interventions to improve quality of life and symptom management, particularly for pain, dyspnea, and fatigue. Palliative care interventions also had strong endorsement. Screening and early detection research should be a priority. It was interesting to see that practice location and highest degree obtained changed the research priorities, which highlights the value of this study since research priorities are often determined by doctors or PhD holders and not other allied-health professionals who have substantial patient-care experience. Of equal importance was what survey respondents did not think should be a research priority, some of which have been the focus of substantial research efforts such as technology to address symptoms and counseling interventions. International societies and funding bodies could consider these topics in their funding decisions and in shaping their strategic directions in the care of patients with lung cancer. These results can also be used as a guide for researchers when thinking about developing research in lung cancer care in a patient-centred research agenda.

## Data Availability Statement

The raw data supporting the conclusions of this article will be made available by the authors, without undue reservation.

## Ethics Statement

The study was reviewed and approved by The Hong Kong Polytechnic University ethics committee. Written informed consent for participation was not required for this study in accordance with the national legislation and the institutional requirements.

## Author Contributions

AM contributed study conception, development of protocol, data analysis, writing paper. AF, MC, PL, DLC, AC contributed to study design and data collection, and provided critical comments to paper drafts.. All authors contributed to the article and approved the submitted version.

## Conflict of Interest

The authors declare that the research was conducted in the absence of any commercial or financial relationships that could be construed as a potential conflict of interest.
